# Hybrid Immunity in a Mozambican Cohort After 1 or 2 Doses of the BBIBP-CorV Vaccine

**DOI:** 10.1093/cid/ciaf095

**Published:** 2025-07-22

**Authors:** Raquel Matavele Chissumba, Gaurav Kwatra, Patrícia Ramgi, Maria Enosse, Adérito Sigaúque, Celso Khosa, Edna Viegas, Odete Bule, José Langa, Nisha Dhar, Christian Mukendi, Denise Langa, Esperança Sevene, Tandile Hermanus, Nelia Manamela, Simone Richardson, Penny Moore, Shabir Madhi, Ilesh V Jani, the SIVCOV study group, Patricia Ramgi, Raquel Matavele Chissumba, Esperança Sevene, Sérgio Chicumbe, Sónia Enosse, Ligia Chambule, Odete Bule, Américo Barata, João Manuel, Nália Ismael, Luísa Namburete, Stefia Vilanculos, Edna Nhacule, Maria Enosse, Adérito Sigauque, Eduardo Namalamgo, Celso Castiano, Mirna Mutombene, Edson Mongo, Ducília Matimbe, Onélia Guiliche, Vania Mapossa, Vania Monteiro, Carmélia Massingue, Edna Viegas, Jose Langa, Ilesh Jani

**Affiliations:** Instituto Nacional de Saúde, Marracuene, Mozambique; Centro de Investigação e Desenvolvimento em Etnobotânica, Namaacha, Mozambique; Department of Clinical Microbiology, Christian Medical College, Vellore, India; South African Medical Research Council, Vaccines and Infectious Diseases Analytics Research Unit, Faculty of Health Sciences, University of the Witwatersrand, Johannesburg, South Africa; Division of Infectious Diseases, Department of Pediatrics, Cincinnati Children's Hospital Medical Center and University of Cincinnati, Cincinnati, Ohio, USA; Instituto Nacional de Saúde, Marracuene, Mozambique; Instituto Nacional de Saúde, Marracuene, Mozambique; Instituto Nacional de Saúde, Marracuene, Mozambique; Instituto Nacional de Saúde, Marracuene, Mozambique; Instituto Nacional de Saúde, Marracuene, Mozambique; Instituto Nacional de Saúde, Marracuene, Mozambique; Instituto Nacional de Saúde, Marracuene, Mozambique; South African Medical Research Council, Vaccines and Infectious Diseases Analytics Research Unit, Faculty of Health Sciences, University of the Witwatersrand, Johannesburg, South Africa; South African Medical Research Council, Vaccines and Infectious Diseases Analytics Research Unit, Faculty of Health Sciences, University of the Witwatersrand, Johannesburg, South Africa; Instituto Nacional de Saúde, Marracuene, Mozambique; Department of Physiological Science, Clinical Pharmacology, Faculty of Medicine, Eduardo Mondlane University, Maputo, Mozambique; South African Medical Research Council Antibody Immunity Research Unit, Faculty of Health Sciences, University of the Witwatersrand, Johannesburg, South Africa; HIV Virology Section, Centre for HIV and STIs, National Institute for Communicable Diseases of the National Health Laboratory Services, Johannesburg, South Africa; South African Medical Research Council Antibody Immunity Research Unit, Faculty of Health Sciences, University of the Witwatersrand, Johannesburg, South Africa; HIV Virology Section, Centre for HIV and STIs, National Institute for Communicable Diseases of the National Health Laboratory Services, Johannesburg, South Africa; South African Medical Research Council Antibody Immunity Research Unit, Faculty of Health Sciences, University of the Witwatersrand, Johannesburg, South Africa; HIV Virology Section, Centre for HIV and STIs, National Institute for Communicable Diseases of the National Health Laboratory Services, Johannesburg, South Africa; South African Medical Research Council Antibody Immunity Research Unit, Faculty of Health Sciences, University of the Witwatersrand, Johannesburg, South Africa; HIV Virology Section, Centre for HIV and STIs, National Institute for Communicable Diseases of the National Health Laboratory Services, Johannesburg, South Africa; South African Medical Research Council, Vaccines and Infectious Diseases Analytics Research Unit, Faculty of Health Sciences, University of the Witwatersrand, Johannesburg, South Africa; Instituto Nacional de Saúde, Marracuene, Mozambique

**Keywords:** hybrid immunity, COVID-19 vaccine, antibodies, inactivated vaccine, BBIBP-CorV

## Abstract

**Background:**

More than half of the BBIBP-CorV vaccines, outside of Pacific Asia, were distributed in Africa. Nevertheless, there are limited data on the immunogenicity of BBIBP-CorV from Africa. We compared the antibody response, after 1 and 2 doses of the BBIBP-CorV vaccine, in individuals seropositive or seronegative to severe acute respiratory syndrome coronavirus 2 prior to vaccination.

**Methods:**

From March to May 2021, blood samples were obtained at first and second doses of the BBIBP-CorV, and 2 weeks later. Antibody titers against the full-length spike, receptor binding domain and nucleocapsid protein (anti-NC) of severe acute respiratory syndrome coronavirus 2 were measured. Pseudovirus neutralization assays and antibody-dependent cellular cytotoxicity (ADCC) against the D614G, BA.2, and BA.4 variants were also evaluated.

**Results:**

At the second dose, the immunoglobulin G titers for full-length spike and anti-nucleocapsid protein, the ADCC against BA-2, and the neutralizing activity against the D614G and BA.2 were higher in individuals seropositive to any of the epitopes at the first dose (n = 26) compared to the levels observed 2 weeks later in the seronegative group (n = 25). We did not observe an increase on magnitude of binding antibodies, ADCC, and neutralizing activities, in those seropositive, after the second homologous dose of the BBIBP-CorV vaccine.

**Conclusions:**

We suggest that 1 dose of the BBIBP-CorV vaccine in seropositive individuals induced better antibodies response including against variant of concerns compared to that observed after 2 doses in seronegative individuals. A further homologous dose of the BBIBP-CorV vaccine, in those who are seropositive, does not improve the antibody response observed after the first dose.

## Teaser

One dose of the BBIBP-CorV vaccine in seropositive individuals induces better antibodies response compared to naïve individuals after 2 doses. A second dose within 3 weeks of the first dose does not further induced antibody response in seropositive individuals.

During the coronavirus disease 2019 (COVID-19) pandemic, 13 COVID-19 vaccines were approved by the World Health Organization for emergency use [1]. Vaccination programs in Africa were established after at least 2 waves of high transmission of SARS-CoV-2, at a stage when several million Africans had experienced natural infection [2].

The whole inactivated virus BBIBP-CorV (Sinopharm, China) was one of the first vaccines to be used in Africa. Overall, 54.8% of the 203 million doses of BIBBP-CorV, outside of Pacific Asia, were delivered to Africa. Nevertheless, there is a paucity of data on the immunogenicity of the BBIBP-CorV, including against Omicron variant of concern (VOC), in African populations.

The BBIBP-CorV vaccine was designed to be administrated on a 2-dose schedule with an interval of 3 weeks between doses [3]. Lower immunogenicity was reported in Egyptians vaccinated with homologous BBIBP-CorV vaccine boost compared with homologous booster with the BNT162b2, ChAdOx1 nCov-19, and Ad26.COV2.S [4, 5].

Several studies have revealed immune advantage among individuals who received mRNA vaccines who were previously exposed to severe acute respiratory syndrome coronavirus 2 (SARS-CoV-2), including against multiple variants of concern, compared to naïve vaccinees [6, 7]. Hybrid immunity induces antibodies with increased potency and breadth [8]. High titers of binding and neutralizing antibodies against SARS-CoV-2 have been associated with risk reduction of COVID-19 disease [9–12]. These titers are also highly predictive of immune protection in convalescent individuals and those who received different COVID-19 vaccines [13].

This study evaluated the effect of antecedent SARS-CoV-2 infection on immune responses induced by 1 and 2 doses of BBIBP-CorV in relation to binding antibodies, magnitude of antibody-dependent cellular cytotoxicity (ADCC) and neutralizing antibody activity against SARS-CoV-2 lineages, including Omicron VOC, in Mozambican health care workers (HCW).

## MATERIAL & METHODS

### Study Participants

**Figure 1.**
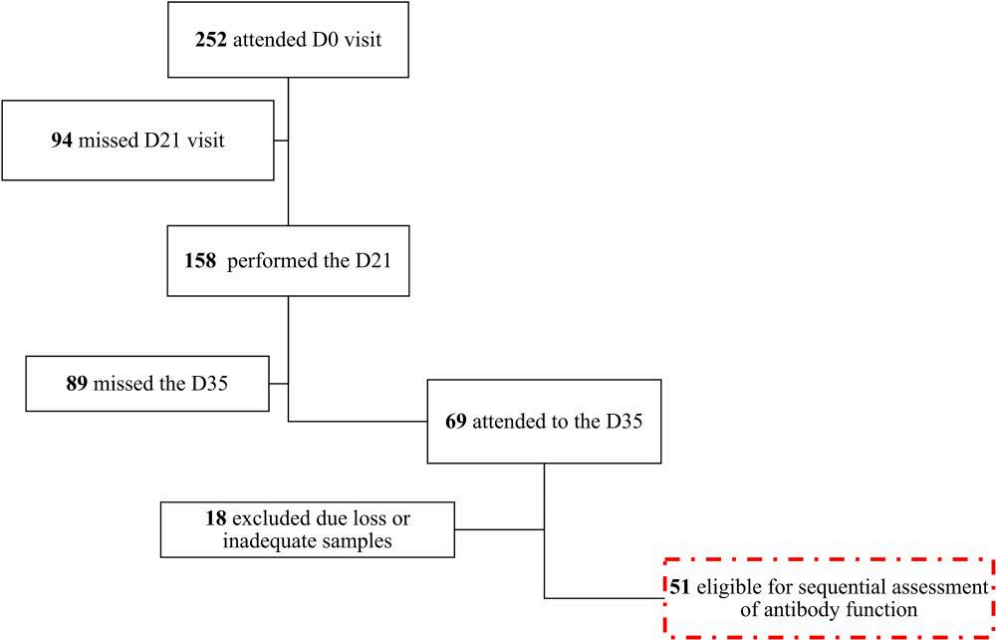
Diagram of the eligibility criteria for selection of participant samples for binding and neutralizing experiments.

The study was conducted from March to May 2021 during the first vaccination campaign and second wave of COVID-19 in Mozambique. Of the 252 HCW initially in the study (SIVCOV) from health facilities offering vaccination in Maputo city, baseline demographic data and human immunodeficiency virus (HIV)-1 status of these participants in the study are shown in Table 1. Peripheral blood samples were collected before the first (D0) and second dose (D21) of BBIBP-CorV, which were given 21 days apart, with a further blood draw being done 2 weeks later (D35). Plasma was separated for blood and stored at −80°C. Inclusion in this study required serum samples from all 3 study time points, which were available from 51 participants (Figure 1). The binding antibody titers were measured at all 3 time points, and ADCC and neutralizing activity against pandemic and Omicron variants was evaluated at D21 and D35. HIV status was also assessed on those who consented.

**Table 1.** Baseline Demographic Data of HCW Seronegative and Seropositive Before BIBBP-CorV Vaccination, based on Reactivity to IgM/IgG Antibodies Against SARS-CoV-2

	Total	Seronegative	Seropositive	*P*
N	252	154	98	
M/F	117/135	89/65	28/70	<.0001
Age (y)				
Median (IQR)	37 [30.0–45.0]	37 [30.0–43.0]	38 [30.3–46.0]	.46
Age groups (y) (n)				
18–30	68	43	25	.71
31–40	96	62	34	
41–50	55	32	23	
51–60	27	14	13	
>60	6	3	3	
Living with HIV-1 (n)	12	5	7	

F, female; HIV-1, human immunodeficiency virus-1; IQR, interquartile range; M, male.

Participants were stratified into 2 groups based on baseline titers of antibodies against the 3 tested proteins. Those with antibody titers below to the cutoff level for all 3 regions of SARS-CoV-2 proteins were classified as seronegative (n = 26) and those with cutoff levels above the cutoff for at least 1 of the tested proteins were classified as seropositive (n = 25).

### Rapid SARS-CoV-2 and HIV-1 Antibodies Testing

The testing for presence of HIV-1 and SARS-CoV-2 antibodies was performed on thawed serum samples, previously stored at −80°C. HIV-1 testing was performed following the national guidelines, with screening performed using the Alere Determine HIV-1/2 (Abbott, USA). Reactive results were confirmed by a second immunochromatographic test, the Trinity Biotech Uni-Gold HIV (Trinity Biotech, Ireland). The screening for antibodies against SARS-CoV-2 was performed using the PANBIO COVID-19 immunoglobulin G (IgG) Rapid Test Device (Abbott, USA).

### Determination of IgG Antibody Titers

Serum samples were analyzed by bead-based assay on Luminex platform for binding antibodies to the full-length spike protein (S), the receptor binding domain (RBD), and the nucleocapsid (NC) region and reported results in binding antibody units per milliliter (BAU/mL) as described [14]. The expression plasmid encoding for S and RBD proteins was obtained from the Florian Krammer, Mount Sinai, USA. The recombinant spike proteins were expressed as described previously [15] and N protein was purchased from BioTech, Africa (Cat no# BA25-P, South Africa). Proteins were coupled to the magnetic microsphere beads (Bio-Rad, USA) using a 2-step carbodiimide reaction [16]. An in-house references serum was developed by pooling convalescent serum from adult who recovered from COVID-19. This reference serum was calibrated against the first World Health Organization international standard for anti–SARS-CoV-2 (NIBSC 20/136) distributed by the National Institute for Standards and Biological Control (NIBSC) to develop and evaluate serological assays for detecting antibodies against SARS-CoV-2 (NIBSC, Potters Bar, UK; https://www.nibsc.org/). Serum samples collected before 2020 (n = 31) were used for analysis of assay specificity. IgG titers of 24 BAU/mL, 29 BAU/mL, and 15 BAU/mL were selected as the thresholds indicative of SARS-CoV-2 seropositivity, based on the highest value of S, RBD, and N protein in samples from pre–COVID-19. Bead fluorescence was read with the Bio-Plex 200 instrument (Bio-Rad) using Bio-Plex manager 5.0 software (Bio-Rad).

### Neutralization Measurements

Pseudovirus neutralization assays were used to measure SARS-CoV-2 neutralizing antibody levels in serum at 21 days after the first dose (D21) and 2 weeks after the second dose (D35). Briefly, 293T/ACE2.MF cells modified to overexpress human angiotensin-converting enzyme 2 and cells were cultured in Dulbecco’s modified Eagle medium containing 10% heat-inactivated fetal bovine serum and 3 μg/mL puromycin at 37°C, 5% CO_2_. The SARS-CoV-2, Wuhan-1 spike, cloned into pCDNA3.1, was mutated using the QuikChange Lightning Site Directed Mutagenesis Kit to include D614G (original) or T19I, L24S, 25-27del, G142D, V213G, G339D, S371F, S373P, S375F, T376A, D405N, R408S, K417N, N440 K, S477N, T478 K, E484A, Q493R, Q498R, N501Y, Y505H, D614G, H655Y, N679 K, P681H, N764 K, D796Y, Q954H, and N969 K (Omicron BA.2). Pseudoviruses were produced by cotransfection with a lentiviral backbone (HIV-1 pNL4.luc encoding the firefly luciferase gene) and either of the SARS-CoV-2 spike plasmids with PEIMAX (Polysicences). Culture supernatants were clarified of cells by a 0.45-μM filter and stored at −70°C. Serum samples were heat-inactivated and clarified by centrifugation. Pseudovirus and serially diluted sera were incubated for 1 hour at 37°C, 5% CO_2_. Cells were added at 1 × 10^4^ cells per well. After 72 hours of incubation at 37°C, 5% CO_2_, luminescence was measured using Perkin Elmer Life Sciences Model Victor X luminometer. Neutralization was measured as described by a reduction in luciferase gene expression after single-round infection of 293T/ACE2.MF cells with spike-pseudotyped viruses. Titers were calculated as the reciprocal plasma dilution (ID50) causing 50% reduction of relative light units (RLU).

### Evaluation of ADCC

The ability of plasma antibodies to cross-link and signal through FcγRIIIa (CD16) and spike expressing cells was measured as a proxy for ADCC. HEK293T cells were transiently transfected with 5 μg of native SARS-CoV-2 spike plasmids (D614G, BA.1, and BA.4) using PEI-MAX 40 000 (Polysciences) and incubated for 2 days at 37°C. AIRU946-A6, which binds to different soluble spike variants, was used to confirm similar amounts of spike expression on the surface of the cells across variants through the detection by anti-IgG phycoerythrin staining measured by flow cytometry. Palivizumab against all variants, untransfected cells, and transfected cells not incubated with monoclonal antibodies were used as negative controls. Subsequently, 1 × 10^5^ spike transfected cells per well were incubated with heat-inactivated plasma (1:100 final dilution) or monoclonal antibodies (final concentration of 100 μg/mL) in RPMI 1640 media supplemented with 10% fetal bovine serum 1% Pen/Strep (Gibco, Gaithersburg, MD) for 1 hour at 37°C. Jurkat-Lucia NFAT-CD16 cells (Invivogen) (2 × 10^5^ cells/well and 1 × 10^5^ cells/well for spike and other proteins, respectively) were added and incubated for 24 hours at 37°C, 5% CO_2_. Twenty microliters of supernatant was then transferred to a white 96-well plate with 50 μL of reconstituted QUANTI-Luc secreted luciferase and read immediately on a Victor 3 luminometer with 1 seconds integration time. Normalized RLU of a no-antibody control was subtracted as background. Palivizumab was used as a negative control, whereas AIRU946-E4 and AIRU946-A6 were used as positive controls showing similar activity across variants. A positive threshold was set using 10 SARS-CoV-2 negative plasma samples before the pandemic. To induce the transgene 1 × cell stimulation cocktail (Thermofisher Scientific, Oslo, Norway), 2 μg/mL ionomycin in R10 was added as a positive control to confirm sufficient expression of the Fc receptor. RLUs for spikes were normalized to each other and between runs using AIRU946-A6. All samples were run head-to-head in the same experiment as were all variants tested. Samples were run in duplicate and repeated a minimum of 2 times.

## STATISTICAL ANALYSIS

Statistical analyses were performed using GraphPad Prism version 9.5.1 (USA). The nonparametric tests, Mann–Whitney, and Kruskal–Wallis test were used for unpaired comparison between 2 and 3 groups, respectively and, Friedman and Wilcoxon paired test for paired comparison among groups. The chi-squared test was used for frequency analyses. Differences or correlations with *P* values < .05 were considered statistically significant.

## RESULTS

### One Dose of the BBIBP-CorV Vaccine Improves the Magnitude of Binding Antibodies Against SARS-CoV-2 in Seropositive Individuals, With No Benefit of a Second Dose

**Figure 2.**
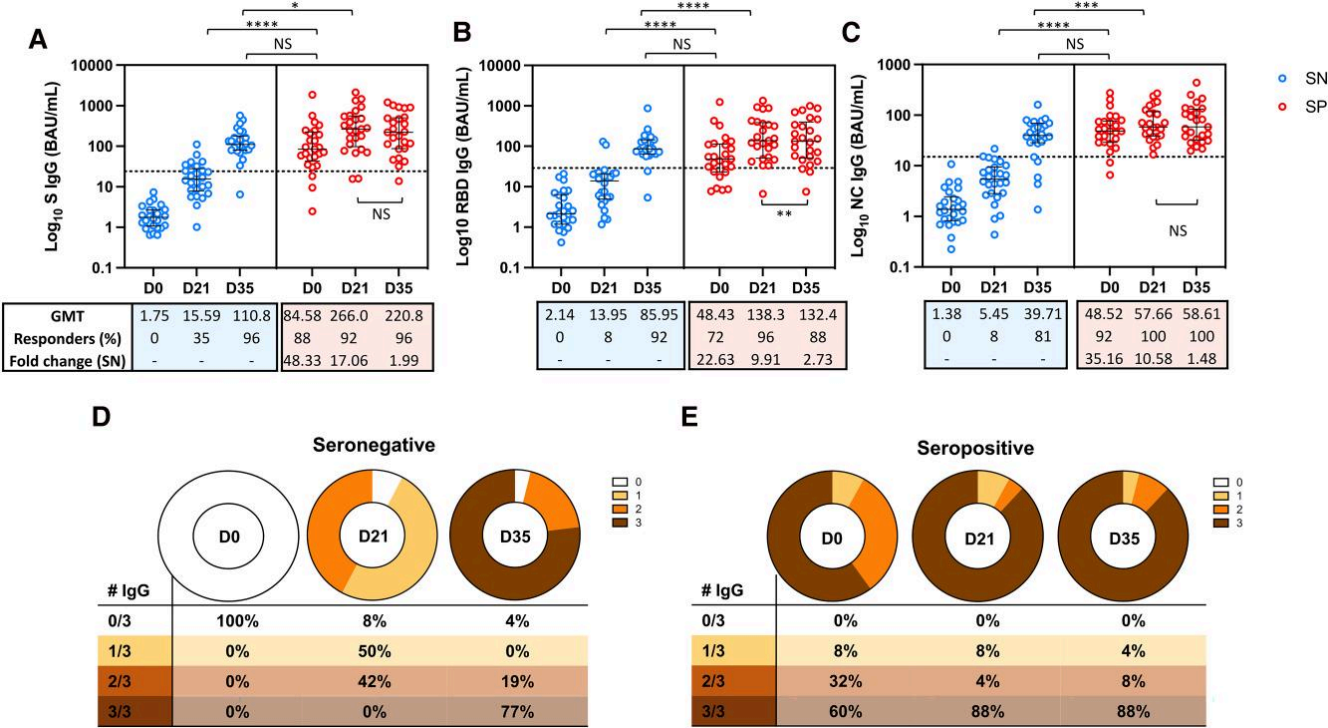
Plasmatic titers of IgG binding antibodies against epitopes of SARS-CoV-2. The median and interquartile ranges of the titers of binding antibodies against the (*A*) S-region, (*B*) RBD, and (*C*) NC region is shown on each graph before the first dose (D0), 21 d after the first dose (D21) and 2 weeks after the second homologous dose (D35) of the BBIBP-CorV vaccine. Each dot represents 1 participant from the seronegative (SN) or seropositive (SP) group. The number of responders on each time point are shown, on each graph, and the cutoff titers for positivity are marked as dot horizontal line on each graph. Significant statistical difference on titers between the time points are showed as *, ***, or **** for *P* values lower that .05, .001, or .0001, respectively.

First, we analyzed the magnitude of binding antibodies before and after 1 or 2 doses for each group. In those who were seronegative, the magnitude of the response against the S, RBD, and N regions of SARS-CoV-2 increased from D0 to D21 (*P* < .0001; *P* < .0001; and *P* < .0001, respectively), and from D21 to 2 weeks after the second dose (D35) (*P* < .0001; *P* < .0001; and *P* < .0001, respectively). In the group that was seropositive, there was an increase in the titers of IgG antibodies targeting the S, RBD, and NC regions, from D0 to D21 (*P* < .0001; *P* < .0001; and *P* < .0001, respectively). However, we did not find a significant difference in the magnitude of IgG antibodies against the S (*P* = .06) and NC (*P* = .92) regions when comparing levels observed at D21 and D35. Furthermore, there was a decrease in the titers of IgG antibodies against the RBD region comparing levels on D21 and 2 weeks later (*P* = .0067) in seropositive participants (Figure 2).

Comparative analysis of the magnitude between the groups seronegative and seropositive at D21 showed that the titers of binding antibodies against the RBD, S, and N regions were 9.91, 17.06, and 10.57, respectively, times higher in the seropositive group compared to the seronegative group of vaccinees (*P* < .0001, *P* < .0001, and *P* < .0001, respectively). These differences between seronegative and seropositive decreased at D35, reaching equivalent titers for RBD (*P* = .37) and S (*P* = .08) regions. However, the titers of the NC protein at D35 were still higher for seropositive compared to seronegative (*P* = .0441).

The median titers of IgG antibodies in the seropositive group at D0 were 3.47, 5.43, and 8.90 times higher compared to those observed in the seronegative group at D21, for the RBD, S, and NC regions, respectively (*P* < .0001, *P* < .0001, and *P* < .0001, respectively). Moreover, the D21 antibodies titers in the seropositive against the S and NC region group were 2.40 and 1.45 times higher than those observed at D35 for the seronegative group (*P* = .0130 and *P* = .0287, respectively) regions. The titers of binding antibodies against the RBD region observed at D21 for the seropositive group were equivalent to those observed at D35 for the seronegative group (138.3 BAU/mL vs 85.95 BAU/mL; *P* = .14).

### One Dose of BBIBP-CorV Vaccine Induces Higher ADCC Activity Against the BA4 Omicron Variants in Seropositive Participants but Levels Drop 2 Weeks After the Second Homologous Dose

**Figure 3.**
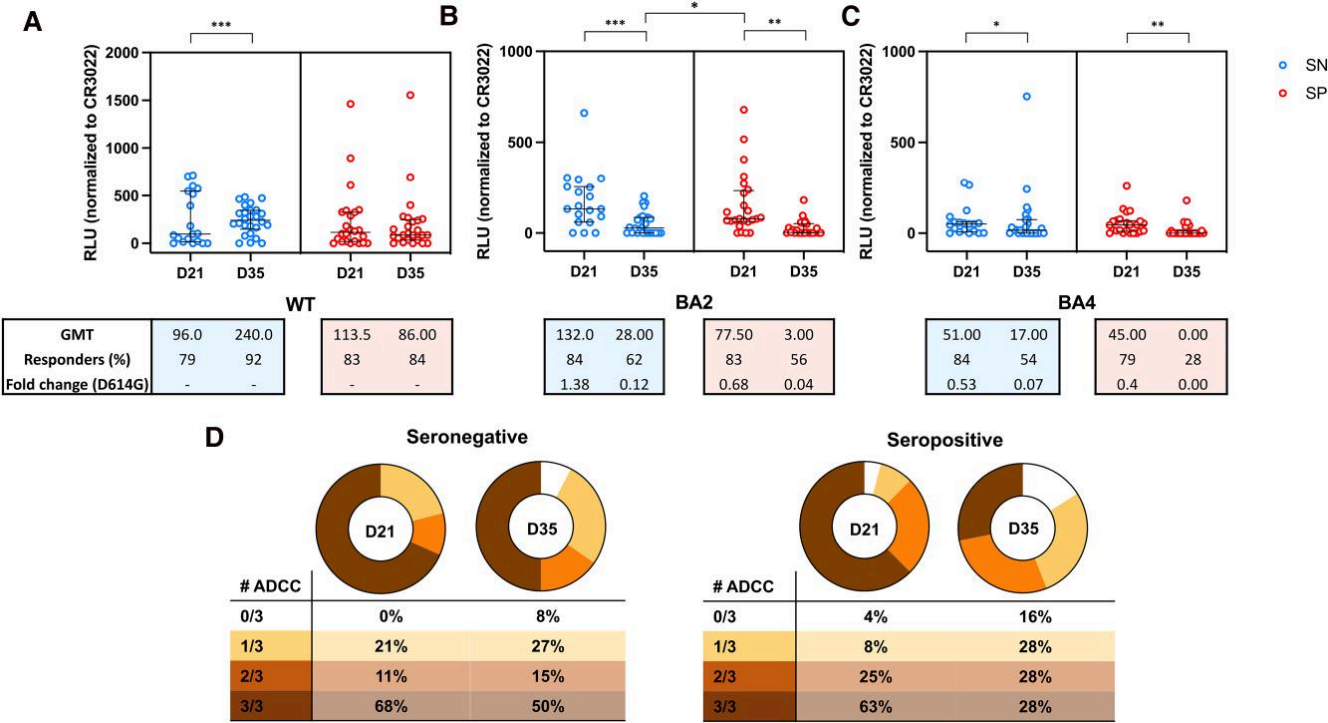
Antibody-dependent cellular cytotoxicity (ADCC) activity against the D614G and Omicron BA.2 and BA.4 sublineages. ADCC activity detected at 21 d after the first dose (D21) and at 2 weeks after the second homologous dose (D35) of the BBIBP-CorV, against (*A*) the pandemic D614G variant (*B*) the BA.2 Omicron sublineage and (*C*) against the BA.4 Omicron sublineage, in seronegative or seropositive participants at time of vaccination. The number of responders on each time point are shown, on each graph, and the cutoff titers for positivity are marked as dot horizontal line on each graph. The median and interquartile ranges of the of ADCC activity against each of the variants, measured as relative light units (RLU), are shown on each graph. Significant statistical difference on RLU between the time points are shown as * or **** for *P* values lower that .05 or .0001, respectively. *D*, Number of participants with detectable ADCC activity at D21 and D35 against 1, 2, or the 3 tested variants.

Furthermore, we analyzed the ADCC response against the wild-type virus (WT; D614G) and against the BA.2 and BA.4 Omicron variants in seronegative and seropositive vaccinated participants at D21 and D35. In terms of dynamics of the response, in the seronegative group, the magnitude of the ADCC against the D614G increased from D21 to D35 (*P* = .0007). However, the ADCC activity against the Omicron variants BA.2 and BA.4 decreased from D21 to D35 (*P* = .0002 and *P* = .0145, respectively). Analysis of the seropositive group revealed no difference on magnitude of ADCC for the D614G variant from D21 to D35 (*P* = .36). However, a decrease in ADCC response was observed for the BA.2 and BA.4 omicron sublineages (*P* = .0020 and *P* = .0049, respectively) (Figure 3).

When performing a comparative analysis between seronegative and seropositive at same visit, we found that the response against the D614G and the BA.4 variants at D21 was higher in seropositive compared to those observed in seronegative (*P* = .0001 and *P* = .0403, respectively). However, no difference between the 2 groups was observed at D35 for these variants. For the BA.2 variant, we did not find differences on the magnitude of the ADCC response between seronegative and seropositive at D21 (*P* = .81) and D35 (*P* = .50).

As observed when analyzing binding antibodies, we found that the magnitude of ADCC response against the BA.2 omicron in seropositive participants at D21 was 2.77 times higher compared to that observed in those seronegative participants at D35 (*P* = .0096). For the BA4 variant and the D614G variant, we did not find differences in magnitude observed for the seropositive participants at D21 compared to the response observed for seronegative at D35.

When we compared the response among the 3 tested variants, we did not observe differences in the magnitude of ADCC activity against D614G and the BA.2 Omicron variant, for both seropositive and seronegative participants at D21. However, the magnitude of ADCC response against the BA4 was lower compared to D614G and the BA2 variants for the seropositive group (*P* = .0009 and *P* < .0001, respectively) and seronegative group (*P* = .0283 and *P* = .0081, respectively). At D35, the magnitude of ADCC response against BA.2 and BA.4 was not significantly different for the both seronegative and seropositive groups. However, it was lower compared to the response against the D614G variant (*P* < .0001 and *P* = .0044 and) and (*P* < .0001 and *P* < .0001) for the seropositive and seronegative groups, respectively.

The magnitude of ADCC activity, particularly against WT, correlated positively with the titers of binding antibodies against RBD and S region observed at D21 and D35 for the seropositive group. For the seronegative group, we also observed that correlation between ADCC activity against the WT virus and the magnitude of binding antibodies against the RBD regions at D35 (Supplementary figure 1).

### One Dose of BBIBP-CorV Vaccine Induces Neutralizing Antibodies Against BA.2 Variant Similar to the D614G Variant in Seronegative, but Levels Drop 2 Weeks After the Second Homologous Dose for the 2 Groups

**Figure 4.**
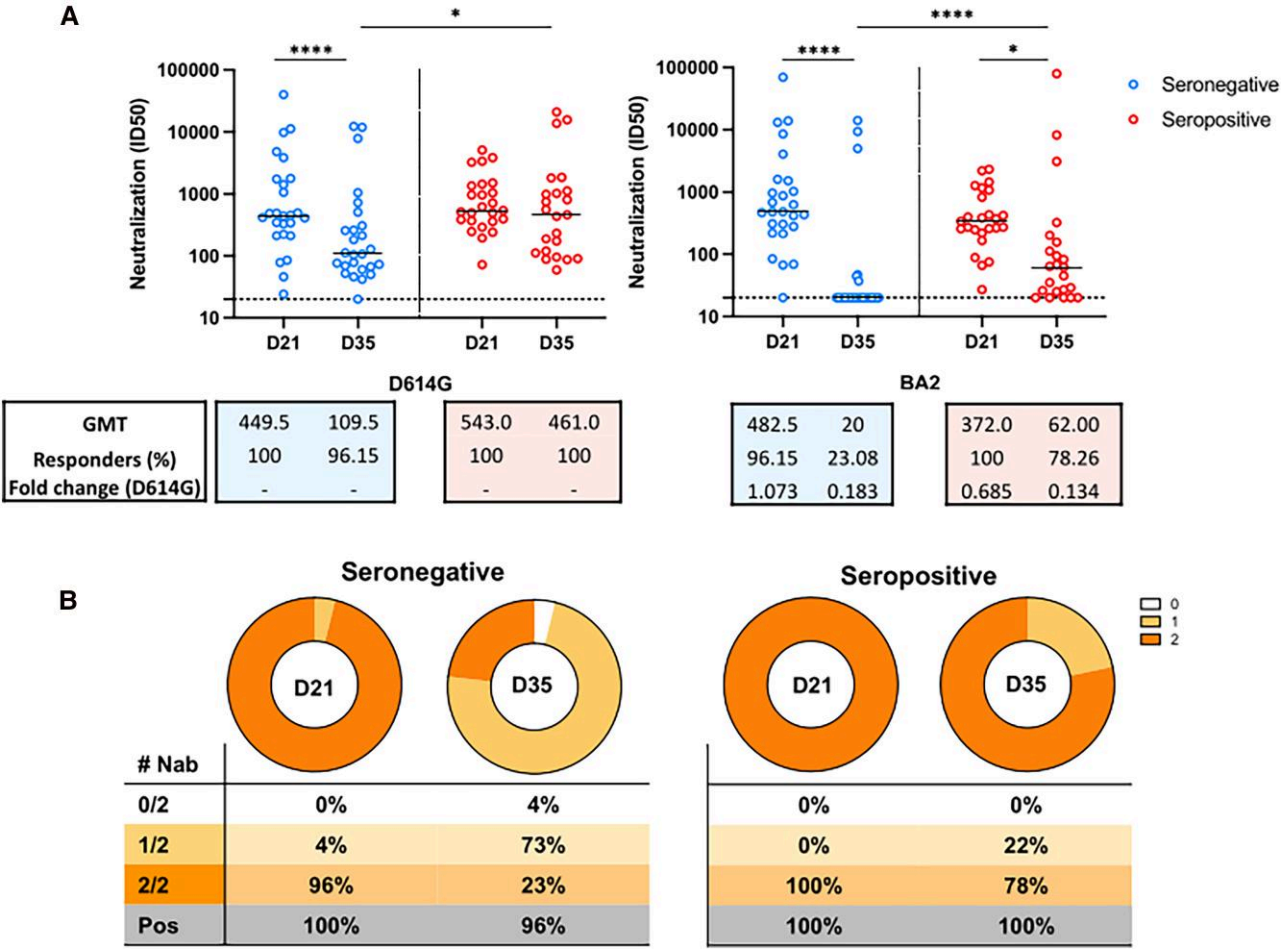
Plasmatic titers of neutralizing antibodies against SARS-CoV-2 variants at 2 weeks after the second homologous dose of the BBIBP-CorV vaccine. *A*, Comparison of the neutralizing titers between those seronegative or seropositive participants before the vaccination against the pandemic D614G variant (left) and Omicron BA2 variant (right). The median and interquartile ranges of the of neutralizing titers against each of the variants are shown on each graph. Significant statistical difference on titers between the time points are shown as * or **** for *P* values lower that .05 or .0001, respectively. The number of responders on each time point are shown, on each graph, and the cutoff titers for positivity are marked as dot horizontal line on each graph. *B*, Number of participants with detectable neutralizing activity at D21 and D35 against 1 or 2 variants, seropositive and seronegative at baseline visit.

We also analyzed the antibodies neutralizing activity against D614G and Omicron BA.2 variants, in seronegative and seropositive participants at D21 and at D35. In seronegative participants, the Nab titers against both the D614G and BA.2 wanned significatively from D21 to D35 (*P* = .0094 and *P* < .0001, respectively). For the seropositive group, we did not observe significant changes on the Nab titers against the D614G from D21 to D35 (*P* = .90). However, for the BA.2, there was a decrease in Nab titers from D21 to D35 (*P* = .0354) in the seropositive group (Figure 4).

We also did a comparative analysis between groups and found that the D614G ID50 neutralizing titers at D21 were not different between the seropositive and seronegative groups (*P* = .48). However, at D35, seropositive participants showed higher titers compared to seronegative participants (*P* = .0418). Similarly, for Nab against the BA.2 variant, there was not a statistical difference between seropositive and seronegative groups at D21 (*P* = .14), but the Nab titers were somehow significatively higher in the seropositive group at D35 (*P* = .0008).

The magnitude of response observed in seropositive participants at D21 against both D614G and BA.2 was significatively higher compared to that observed for seronegative participants at D35 (*P* = .0004 and *P* < .0001, respectively).

Moreover, for those who were seropositive, the ID50 against the D614G strain was higher compared to that observed for the BA.2 strain at D21 (*P* = .0136) and D35 (*P* = .0008). For the seronegative group, we did not observe differences in the magnitude of the neutralizing activity against the D614G and the Omicron BA.2 at D21. However, at D35, the neutralizing activity against the pandemic virus D614G in the seronegative group was higher compared to the Omicron variant (*P* = .0012).

We did not find a significative correlation between the magnitude of neutralizing activity observed against the pandemic virus D614G and the Omicron BA.2 at D21 and D35 and the titers of binding antibodies against any of the tested regions in both seronegative and seropositive groups (Supplementary figure 1).

## DISCUSSION

Our results suggests that 1 dose of the BBIBP-CorV vaccine in seropositive individuals induces better antibody response compared to 2 doses of BBIBP-CorV in naïve individuals. This hybrid immunity is characterized by improvement in terms of binding, ADCC, and neutralizing activity against SARS-CoV-2 lineages. However, a second homologous dose with the BBIBP-Cor vaccine provides further improvements on magnitude of these vaccine-induced responses in seropositive individuals, consistent with serological data from mRNA vaccines [17–19].

Lower efficacy and immunogenicity of inactivated vaccines when compared to other types of vaccines have been reported elsewhere [4, 20, 21]. However, little information on the immunogenicity of inactivated vaccines in populations of southern Africa has been published, including the magnitude of the escape of Omicron subvariants to vaccine-induced antibodies.

We reported the prevalence of SARS-CoV-2 antibodies in the general population and on different occupational groups during the first wave of COVID-19 in Mozambique [22]. In Maputo, the prevalence of SARS-CoV-2 antibodies in the general population was higher than those observed in HCW, estimated as 2.6%. Here, we found that, after the peak of the second wave dominated by the B.1.351 variant, 39% of the HCW in Maputo presented antibodies against SARS-CoV-2 before vaccination. This high prevalence of exposure to natural infection during a pandemic highlights the relevance of understating the effect of vaccination in populations with multiple exposures to infection in a context of limited vaccine availability.

Analysis of the reinfection risk in individuals with 1 or 2 doses of hybrid immunity and in those with natural infection showed that hybrid immunity was associated with a lower risk of COVID-19 hospitalization than natural immunity [23]. Furthermore, in an increase on ADCC activity and Nab response against the pandemic, Beta and Delta variants are reported in individuals vaccinated with prior exposure [24]. Studies performed in individuals with COVID-19 showed that the ADCC response in patients who recovered from severe disease was stronger than the ADCC from those who died [25]. Moreover, ADCC response induced by natural infection was stronger and longer lasting than that induced by mRNA vaccine [26].

Here, we observed that vaccination with 1 dose of BBIBP-CorV induced modest ADCC response against the pandemic and Omicron variants BA.2 and BA4 in most of the participants. We also observed a stronger ADCC against the WT and BA4 Omicron variants in seropositive individuals after 1 vaccine dose compared to that observed in seronegative participants. A modest ADCC induction in response to the pandemic variant and Omicron sublineages was observed after vaccination with different approaches, including the BBIBP-Cor and other inactivated vaccines [27]. Modest ADCC response against different SARS-CoV-2 variants was also observed after 1 dose of Ad26.COV2 or BNT162b2 [24, 28, 29]. In these cases, exposure to the virus before or after vaccination boosted the response [24, 27–30].

During natural infection, the number of RBD-specific memory B and the neutralizing activity of antibodies against the RBD of SARS-CoV-2 remains stable between 6 and 12 months after infection [8]. Following vaccination with BNT162b2 mRNA vaccine, the repertoire of B cell–producing neutralizing antibodies in individuals previously exposed to SARS-CoV-2, respond to vaccination high titers of neutralizing antibodies that are not susceptible to escape variants than naïve individuals [31]. Our analysis here on vaccination with BBIBP-CorV vaccine, in a period dominated by the B.1.351 variant, corroborate these findings, showing higher titers of binding and neutralizing antibodies against the pandemic and the Omicron variants after 1 vaccine dose in exposed individuals in relation to those naïve at time of the vaccination. This broad response has been associated to ongoing antibody somatic mutation, clonal turnover of memory B cell due to prolonged or repeated antigen exposure leading to the development of monoclonal antibodies resistant to mutation on SARS-CoV-2 RBD, including those found in the variants of concern [8, 32]. Despite the low immunogenicity reported for inactivated vaccines, we observed that almost all subjects that were naïve at the time of vaccination developed Nab against BA.2 variant and the WT virus after 1 dose of the BBIBP-Cor, contrary to the 50% respondents for the WT virus reported in the context of mRNA vaccines [18]. However, neutralizing activity against variants of concern that were similar to or greater than those against the original Wuhan Hu-1 strain was also observed in individuals vaccinated with an RNA vaccine [33, 34].

Although we found some correlation between the magnitude of binding antibodies against S and RBD region and ADCC response, particularly in seropositive subjects, no correlation was seen between the magnitude of binding antibodies against the S, RBD, and NC regions and the neutralizing activity against the pandemic or Omicron variants, in both seronegative and seropositive participants. Independence in terms of postvaccination serum antibody levels and memory B cells or between ADCC and neutralization activity was also reported, particularly for naïve participants after 1 dose of SARS-CoV-2 mRNA vaccines [18, 29].

We also observed that after 2 doses of the inactivated BBIBP-CorV vaccine in individuals seropositive at time of vaccination, there was a significant decrease of IgG antibodies titers against the S region. A rapid wane or absence of improvement after the second dose was also observed in the magnitude of ADCC responses and on neutralizing function against the pandemic and Omicron variants, particularly in those seropositive before vaccination. Absence of improved response in terms of antibodies titers against the RBD and S region, and on memory B-cell frequency 1 week after a second homologous but with mRNA vaccines were also reported [18].

In individuals with HIV, part of the observed failure in inducing the desired responses after vaccination have been also attributed to multiple boosters of immunization that causes an immune attrition [35]. These results resemble the outcome of experiments on Balb/c mice showing that extended vaccination with RBD boosters abrogated the protective immune memories by promoting adaptive immune tolerance [36]. Thus, a second dose in our cohort, of individuals with seropositivity to SARS-CoV-2 before vaccination could also induce a state of immune tolerance. A study conducted in Argentina in HCW who received the inactivated BBIBP-CorV vaccine also found that higher antibodies levels were reached after a single dose, with no evidence of increased response after the second dose [37]. The same 1-dose benefit was observed in other COVID-19 vaccination regimens [18, 33, 38, 39]. It was suggested that the potency of ADCC at some extent is determined by the induction of antibodies recognizing a broad range of spike epitopes [30]. Thus, the difference in outcomes, in this case for ADCC, could be also related by the poor induction of anti-S antibodies following vaccination with BBIBP-CorV compared to other approaches.

It is also important to note that in most of the studies reporting the use of BBIBP-CorV, the analysis was performed at different time points as in this study, and the data were analyzed considering heterologous approaches that included the BBIBP-CorV vaccine. It has been suggested that longer intervals between vaccine doses may induce more robust responses than standard protocols, particularly in those without prior exposure [40, 41]. Heterologous approaches, used widely for HIV-1 vaccines and currently employed for COVID-19 vaccines, would probably improve the vaccine immunogenicity [42–44] and overcome the waning or redundance of antibody titers seen in those exposed, as observed in our cohort.

Overall, despite the small sample size of the study, high rate of loss of follow-up and very limited information of the medical history of the study participants, our results suggest that inactivated vaccines induce modest antibody responses, including against VOC. Furthermore, these findings reinforce the need of continuous improvement of inactivated vaccine formulations to respond the emergence of new variants. A second dose of the BBIBP-CorV within 3 weeks of the first dose not increase titers of binding antibodies nor their effector functions, particularly in individuals with previous SARS-CoV-2 infection. During pandemics of highly transmissible viruses and in contexts of limited vaccine availability, immunization with 1-dose schedules may induce sufficient immunity in populations previously exposed to natural infection.

## Supplementary Material

ciaf095_Supplementary_Data

## Supplementary Data

Supplementary materials are available at *Clinical Infectious Diseases* online. Consisting of data provided by the authors to benefit the reader, the posted materials are not copyedited and are the sole responsibility of the authors, so questions or comments should be addressed to the corresponding author.

## Notes

***Acknowledgments.*** We are thankful to all participants who made this work possible. Special thanks go to the institutions that supported this work including the Instituto Nacional de Saúde (Mozambique), the Faculty of Health Sciences at the University of the Witwatersrand (South Africa), and the National Institute for Communicable Diseases (South Africa). The authors are also thankful to the study participants who made this work possible. The SIVCOV Study Group includes: Patricia Ramgi, Raquel Matavele Chissumba, Esperança Sevene, Sérgio Chicumbe, Sónia Enosse, Ligia Chambule, Odete Bule, Américo Barata, João Manuel, Nália Ismael, Luísa Namburete, Stefia Vilanculos, Edna Nhacule, Maria Enosse, Adérito Sigauque, Eduardo Namalamgo, Celso Castiano, Mirna Mutombene, Edson Mongo, Ducília Matimbe, Onélia Guiliche, Vania Mapossa, Vania Monteiro, Carmélia Massingue, Edna Viegas, Jose Langa, and Ilesh Jani.

***Authors contributions.*** R.M.C., P.R., J.L., S.M., G.K., E.V., C.K., D.L., P.M., and I.J. designed the study. G.K., C.M., N.D., A.S., M.E.­­­­, S.R., and T.H. performed the experiments. R.M.C., P.R., M.E., and O.B. participated in study coordination. D.L. provided supervision and project administration. R.M.C. performed the data analysis, interpretation, and drafted the manuscript. All authors reviewed the manuscript, provided feedback, and approved of the manuscript in its final form.

***Data availability.*** The datasets used and analyzed during the current study are available from the corresponding author on reasonable request.

***Ethics approval and consent to participate.*** All subjects gave their informed consent for inclusion before they participated in the study. The study was conducted in accordance with the Declaration of Helsinki, and the protocol was approved by the Ethics Committee of 36/CNBS/2021. All participants signed the informed consent form to participate in this study and all methods were carried out in accordance with relevant guidelines and regulations.

***Financial support.*** This work was supported by Bill and Melinda Gates Foundation #INV-033882. Under the grant conditions of the Foundation, a Creative Commons Attribution 4.0 Generic License has already been assigned to the Author Acceptable Manuscript version that might arise from this submission. The funding sources was not involved in study design, data collection, analysis, interpretation of data, writing the manuscript nor in decision to submission of the article for publication. The opinions or assertions contained herein are the private views of the author, and are not to be construed as official, or as reflecting true views of the affiliated institutions.

***Supplement sponsorship.*** This article appears as part of the supplement “COVID-19 Vaccine Research in Africa,” sponsored by the International Vaccine Institute and funded by the Coalition for Epidemic Preparedness Innovations (CEPI).
