# Supplementary material for: Hybrid Immunity in a Mozambican Cohort After 1 or 2 Doses of the BBIBP-CorV Vaccine
Source: Clin Infect Dis. 2025 Jul 22;80(Suppl 1):S57–65. doi: 10.1093/cid/ciaf095 (PMC12282517; doi:10.1093/cid/ciaf095)
Supplement: ciaf095_Supplementary_Data [file ciaf095_supplementary_data.pptx]

## Slide 1
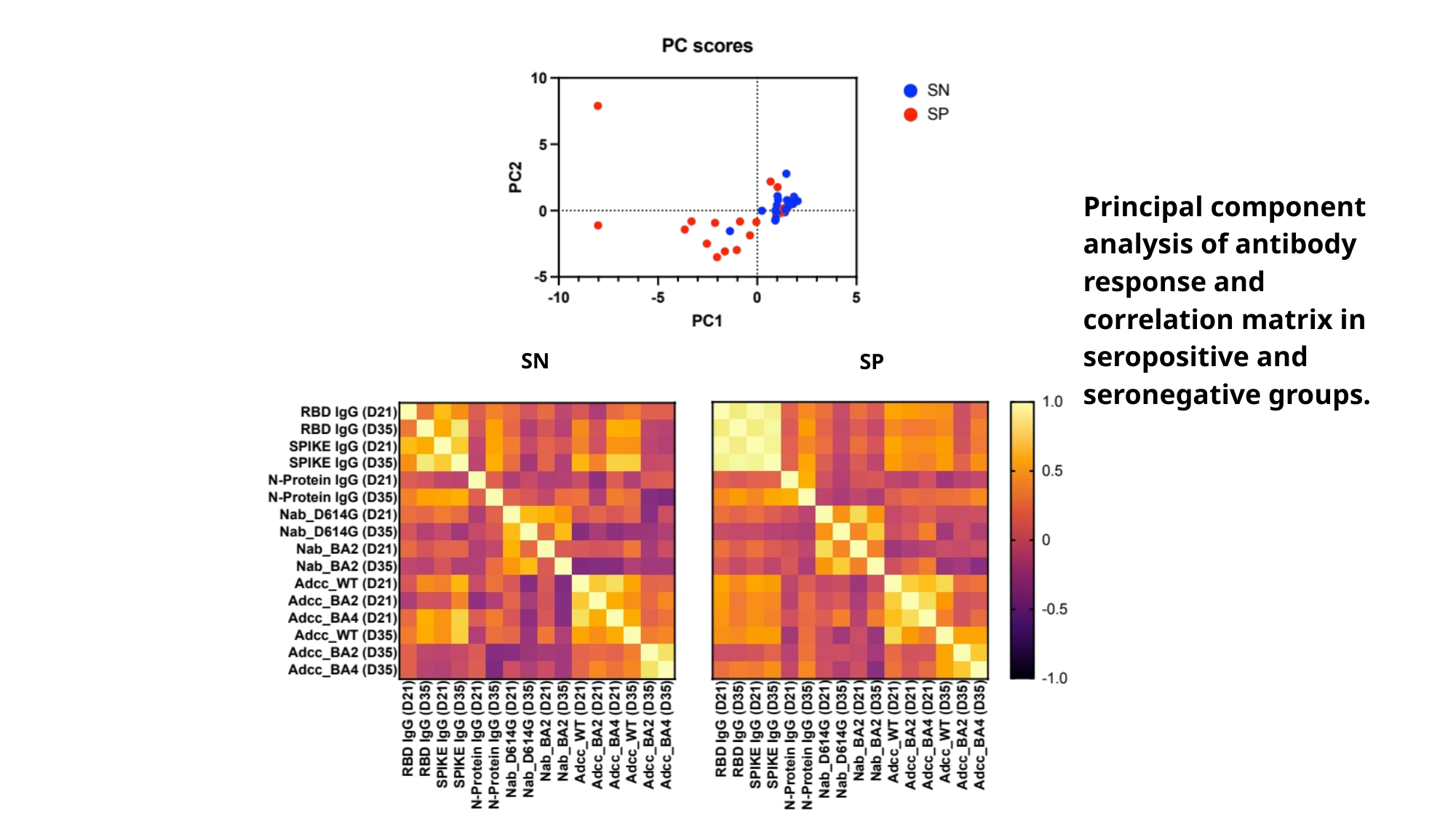

Principal component analysis of antibody response and correlation matrix in seropositive and seronegative groups.
SN
SP
